# Patient-specific resurfacing implant knee surgery in subjects with early osteoarthritis results in medial pivot and lateral femoral rollback during flexion: a retrospective pilot study

**DOI:** 10.1007/s00167-021-06749-8

**Published:** 2021-10-03

**Authors:** Philippe Moewis, René Kaiser, Adam Trepczynski, Christoph von Tycowicz, Leonie Krahl, Ansgar Ilg, Johannes Holz, Georg N. Duda

**Affiliations:** 1 Berlin Institute of Health at Charité-Universitätsmedizin Berlin, Julius Wolff Institute, Augustenburger Platz 1, 13353 Berlin, Germany; 2 OrthoCentrum Hamburg, Hansastrasse 1-3, 20149 Hamburg, Germany; 3 Zuse Institute Berlin, Berlin, Germany

**Keywords:** Metallic resurfacing implants, Episealer implants, Osteoarthritis, Focal chondral lesions, Osteochondral lesions, UKA, TKA, Knee kinematics, Fluoroscopy

## Abstract

**Purpose:**

Metallic resurfacing implants have been developed for the treatment of early, small, condylar and trochlear osteoarthritis (OA) lesions. They represent an option for patients who do not fulfill the criteria for unicompartmental knee arthroplasty (UKA) or total knee arthroplasty (TKA) or are too old for biological treatment. Although clinical evidence has been collected for different resurfacing types, the in vivo post-operative knee kinematics remain unknown. The present study aims to analyze the knee kinematics in subjects with patient-specific episealer implants. This study hypothesized that patient-specific resurfacing implants would lead to knee kinematics close to healthy knees, resulting in medial pivot and a high degree of femoral rollback during flexion.

**Methods:**

Retrospective study design. Fluoroscopic analysis during unloaded flexion–extension and loaded lunge was conducted at > 12 months post-surgery in ten episealer knees, and compared to ten healthy knees. Pre- and post-operative clinical data of the episealer knees were collected using a visual analog scale (VAS), the EQ 5d Health, and the Knee Injury and Osteoarthritis Outcome Score (KOOS) questionnaires.

**Results:**

A consistent medial pivot was observed in both episealer and healthy knees. Non-significant differences were found in the unloaded (*p* = 0.15) and loaded (*p* = 0.51) activities. Although lateral rollback was observed in both groups, it was significantly higher for the episealer knees in both the unloaded (*p* = 0.02) and loaded (*p* = 0.01) activities. Coupled axial rotation was significantly higher in the unloaded (*p* = 0.001) but not in the loaded (*p* = 0.06) activity in the episealer knees. Improved scores were observed at 1-year post-surgery in the episealer subjects for the VAS (*p* = 0.001), KOOS (*p* = 0.001) and EQ Health (*p* = 0.004).

**Conclusion:**

At 12 month follow-up, a clear physiological knee kinematics pattern of medial pivot, lateral femoral rollback and coupled axial external femoral rotation during flexion was observed in patients treated with an episealer resurfacing procedure. However, higher femoral rollback and axial external rotation in comparison to healthy knees was observed, suggesting possible post-operative muscle weakness and consequent insufficient stabilization at high flexion.

## Introduction

Around 80% of knee joint surgeons have identified a “treatment gap” for active patients with focal chondral or osteochondral lesions but otherwise intact knee joints [12, 24]. Most of these patients, although relatively young, have surpassed the age for biological treatment (e.g., autologous chondrocyte transplantation) [1, 10, 26, 28]. On the other hand, total knee arthroplasty (TKA) is not considered a viable option for these patients [6, 12, 17] and unicompartmental knee arthroplasty (UKA) should be reserved for bone-to-bone disease and not for focal chondral or osteochondral lesions [5, 6, 8, 21].

To address such patient needs, metallic resurfacing implants have been developed for the treatment of focal, small, condylar and trochlear osteoarthritis (OA) lesions [2, 7, 13, 16]. A prospective study conducted by Dhollander et al. showed a gradual clinical improvement in time but also significant radiographic changes during a follow-up period of 2–3 years [3]. Laursen et al. found improved subjective outcome as well as reduced pain [9] but also a concerning 23% re-operation rate [11].

Patient-specific resurfacing implants (Episealer^®^) have been developed considering the lesion-size as well as the patient anatomy [10]. Animal studies have shown a firm and consistent bond of the implant to the surrounding bone [14, 16]. Two recent studies showed significant clinical improvement at 24 month post-surgery, good implant safety and low failure rate of 2.5% [6, 15]. Although clinically proven, necessary information about the post-operative alteration of in vivo knee joint kinematics in comparison to healthy knees is missing. Such information could offer new perspectives during the decision-making process prior to knee surgery. It could also facilitate possible predictions on kinematic outcomes after resurfacing implants surgery.

Analyses of healthy knees have shown that a specific degree of femoral lateral rollback, medial pivot and coupled external femoral rotation appears to be essential to enable deep flexion and to avoid excessive shear in the patellofemoral joint [23].

Due to the reduced invasiveness of a partial, focal reconstruction, this study hypothesized that patient-specific resurfacing implants would lead to knee kinematics close to healthy knees, resulting in medial pivot and a high degree of femoral rollback during flexion.

## Materials and methods

### Patients

**Fig. 1:**
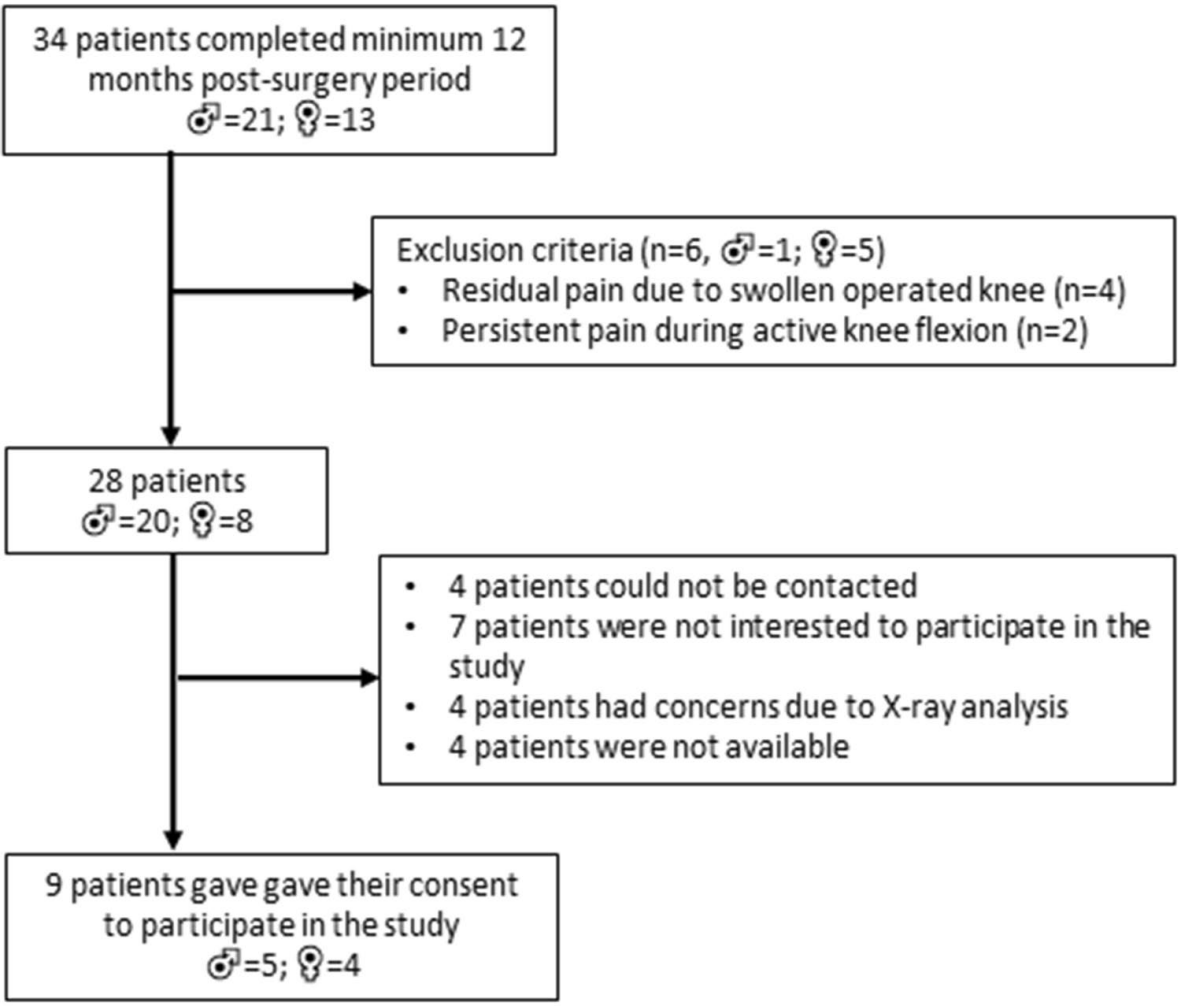
CONSORT diagram describing the recruitment procedure of patients with episealer implants

In a retrospective study, ten knees (from nine patients with one patient treated on both knees; demographics in Table 1) were treated with Episealer^®^ implants (Episurf Medical, Stockholm, Sweden) and recruited for kinematic analysis. Of the ten knees, seven were treated with an Episealer^®^ Solo implant (six on the medial femoral condyle and one on the lateral femoral condyle), two were treated with an Episealer^®^ Trochlea Solo implant, and one received both trochlear and medial femoral condyle implants. The nine recruited patients were selected from an original pool of 34 patients treated with episealer implants, who have completed a minimum of twelve months post-surgery and fulfilled the inclusion criteria of no additional knee surgery. Six patients could not fulfill the second inclusion criteria of no post-operative knee joint pain. Additional reasons for exclusion are summarized in the CONSORT diagram in Fig. 1. No signs of extension/flexion deficits were identified in the episealer subjects. Also, no complications or revision occurred in the period between surgical procedure and data collection. Demographic data on the 9 recruited and measured episealer patients are provided in Table 1. Additionally, a comparison of the demographic data between the 9 recruited and the 25 excluded episealer patients is provided in Appendix Table 5.

**Table 1:** Demographic data of the episealer and healthy subjects

	Sex	Age (years)	BMI	Extension (pre) (°)	Extension (post) (°)	Passive flexion (pre) (°)	Passive flexion (post) (°)		Sex	Age (years)	BMI	Extension (°)	Passive flexion (°)
Episealer 01	m	52	30.6	0	0	120	130	Healthy 01	m	47	26.1	0	140
Episealer 02/03	f	48	21.9	0	0	130	130	Healthy 02	f	48	19.7	0	146
Episealer 04	f	46	22.6	0	0	100	120	Healthy 03	m	18	25.4	0	140
Episealer 05	f	57	25.9	0	0	130	130	Healthy 04	m	38	24.8	0	146
Episealer 06	f	73	26.0	0	0	110	130	Healthy 05	m	35	28.0	0	120
Episealer 07	m	54	36.1	0	0	130	130	Healthy 06	m	43	29.3	0	140
Episealer 08	m	59	32.3	0	0	130	130	Healthy 07	m	35	21.4	0	140
Episealer 10	m	56	38.9	0	0	120	130	Healthy 08	m	46	34.2	0	120
Episealer 11	m	61	29.3	0	0	130	140	Healthy 09	f	24	19.8	0	145
Mean ± SD		56.2 ± 7.9	29.3 ± 5.8			122.2 ± 10.9	130.0 ± 5.0	Healthy 10	m	35	28.0	0	140
*p* value (episealer pre/post)						0.02		Mean ± SD		35.8 ± 9.8	25.6 ± 4.5		137.7 ± 9.6
								*p* value (episealer post/healthy)		0.001	0.14		0.06

Significant differences were observed for passive flexion between the pre- and post-operative states in the episealer subjects. Despite significant differences in age, non-significant differences were observed between episealer (post-operative) and healthy subjects for BMI and passive flexion

The Episealer^®^ implants were manufactured from cobalt chrome with a highly polished articular surface. The implant design was customized and based on an MRI scan and an associated pre-operative planning of the reconstruction of the focal lesion, such that an optimal lesion coverage and patient-specific implant surface (e.g., 3D curvature) replicated the degenerated articular surface. The implant backside (undersurface) was coated with titanium and hydroxylapatite to guarantee an adequate bony integration and thus fixation of the implant.

Ten healthy knees (demographics in Table 1) that were previously measured and analyzed under the same conditions were selected from the Julius Wolff Institute database for comparison. Previous X-ray analysis showed no signs of OA or extension/flexion deficits in the healthy knees.

To provide a different point of comparison apart from the main aim of the study, a set of earlier TKA cases was also selected from the Julius Wolff database. These represented 20 cases of TKA implants with a gradually changing femoral radius design [G-Curve, cruciate retaining (CR) rotating platform, demographics in Appendix Table 6] and ten cases of TKA implants with a conventional femoral multi-radius design (J-Curve, CR, rotating platform, demographics in Appendix Table 6). Inclusion criteria were a primary diagnosis of osteoarthritis with coronal deformity < 10° and no previous open knee surgery. These TKA implant designs were previously analyzed under identical conditions used for the current episealer reconstructed designs.

### Surgical procedure in episealer implants

Treatment with episealer implants was indicated for patients with symptomatic grade III and IV chondral and osteochondral defects in the knee with previous failed conservative treatment and who were suitable for the procedure as it was determined on specific MR images and satisfactory mapping according to an individualized damage marking report. Contraindications included patients with inflammatory arthritis, age below 35 years or above 70 years, malalignment > 5 degrees, joint space narrowing on weight-bearing X-rays and greater than 50% loss of meniscal tissue [6]. All patient-specific episealer designs were based on detailed MRI scans which included four two-dimensional (2D) diagnostic sequences and one three-dimensional (3D) sequence to allow for a 3D computer reconstruction of distal femoral bone and cartilage. The set of surgical instruments consist of six pieces, two of which were individualized: the Epiguide and the Epidummy. Additional details on reconstruction of specific episealer designs, guide instrumentation and surgical procedure have been summarized previously [6, 13]. The post-operative protocol included protected touch weight-bearing during 2 weeks followed by progressive full weight-bearing over the subsequent 2 weeks. Full range of motion was allowed from the outset. Patients were advised not to return to impact type sports. Additional details on post-operative protocols have been provided previously [6].

### Data acquisition at a minimum of 12 months post-surgery

Single-plane X-ray fluoroscopy analysis was performed at the Julius Wolff Institute, Charité-Universitätsmedizin using a Philips BV Pulsera device (Philips Medical Systems GmbH, Hamburg, Germany). The device was adjusted to acquire X-ray images at 30 Hz, 8 ms pulse width, beam energy 60 kVp, beam current 5 mA. Image resolution was 1024 × 1024 pixels with a 12-bit color depth. Additional images of a Perspex calibration box were collected to correct for image distortion [4, 19, 22]. Two activities, single leg weight-bearing lunge and single leg unloaded knee flexion–extension, were selected to analyze the magnitude of femoral rollback during challenging knee flexion. Both activities were carefully explained in advance to ensure that they could be conducted properly by the patients to limit exposure to X-ray radiation during activities.

**Fig. 2:**
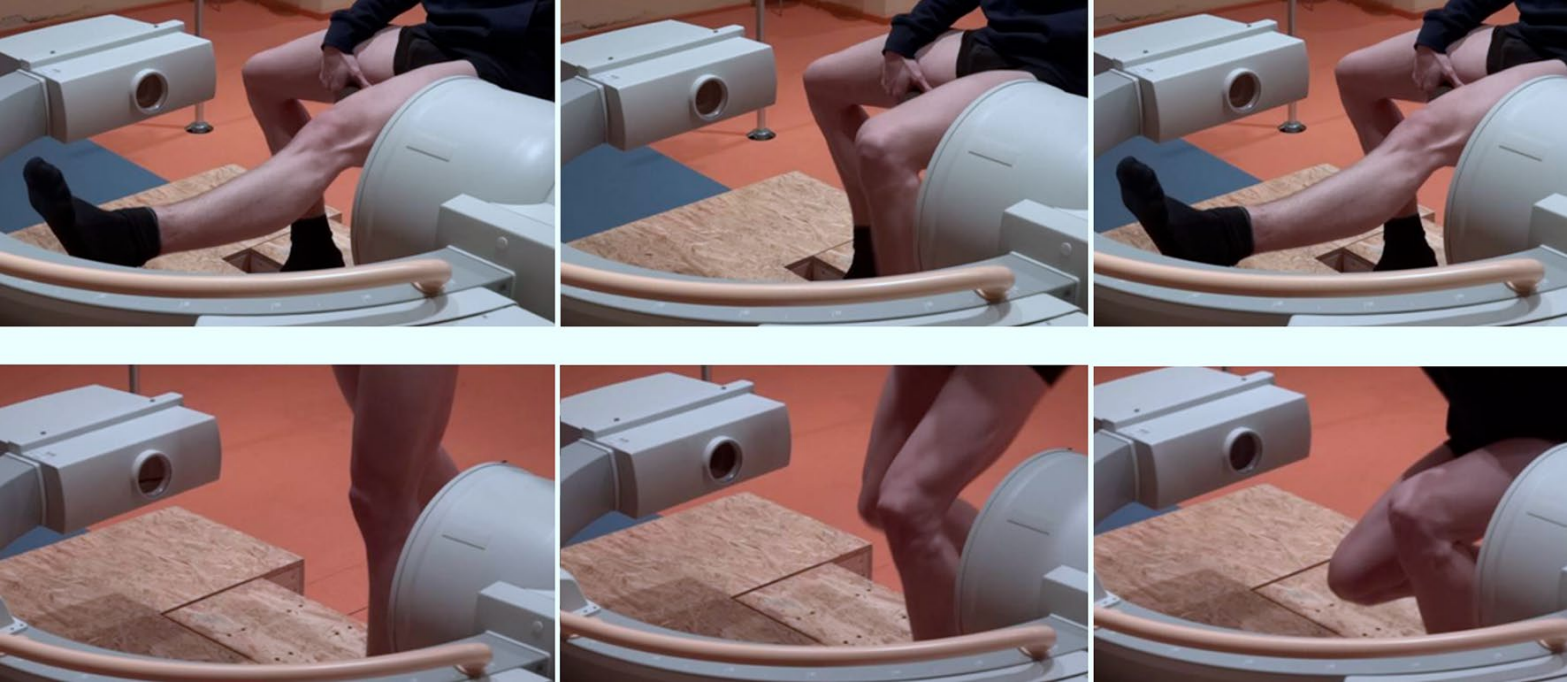
Top: unloaded knee flexion–extension. Bottom: weight-bearing lunge

The lunge activity was conducted with both feet at the same level on a platform. The activity started at full knee extension followed by maximal knee flexion and finished after returning to full knee extension (Fig. 2). The contralateral leg was positioned posteriorly to avoid overlapping. The flexion–extension activity, which was performed seated, started at full knee extension, followed with maximal knee flexion and returned back to full knee extension. For each activity, three repetitions were collected for each knee [19, 22, 25]. Considering the acquisition frequency of 30 Hz and the varied duration of the activity (8–15 s), between 250 and 450 frames were collected during each repetition.

### Clinical data and questionnaires

Pre- and post-operative clinical data of the episealer knees were collected using a visual analog scale (VAS), the EQ 5d Health Questionnaire and the Knee Injury and Osteoarthritis Outcome Score (KOOS).

### Data post-processing and analysis

The collected “Digital Imaging and Communications in Medicine” (DICOM) were divided into single X-ray images. The specific images from maximal extension to maximal flexion were selected in intervals of 5° of flexion for all repetitions for both activities.

Patient-specific femoral and tibial bone surfaces were generated by manual segmentation using Mimics (Materialise NV) from the collected MRI scans. The reconstructed 3D surfaces were registered to the selected fluoroscopic images in a procedure previously described [20]. The registration is based on automatic contour detection followed by manual corrections to select and discard the erroneous contours. The accuracy of this procedure has been analyzed previously under dynamic conditions, with reported root-mean-square error values of 0.2–0.6 mm for translations and 0.4°–0.8° for rotations [20].

**Fig. 3:**
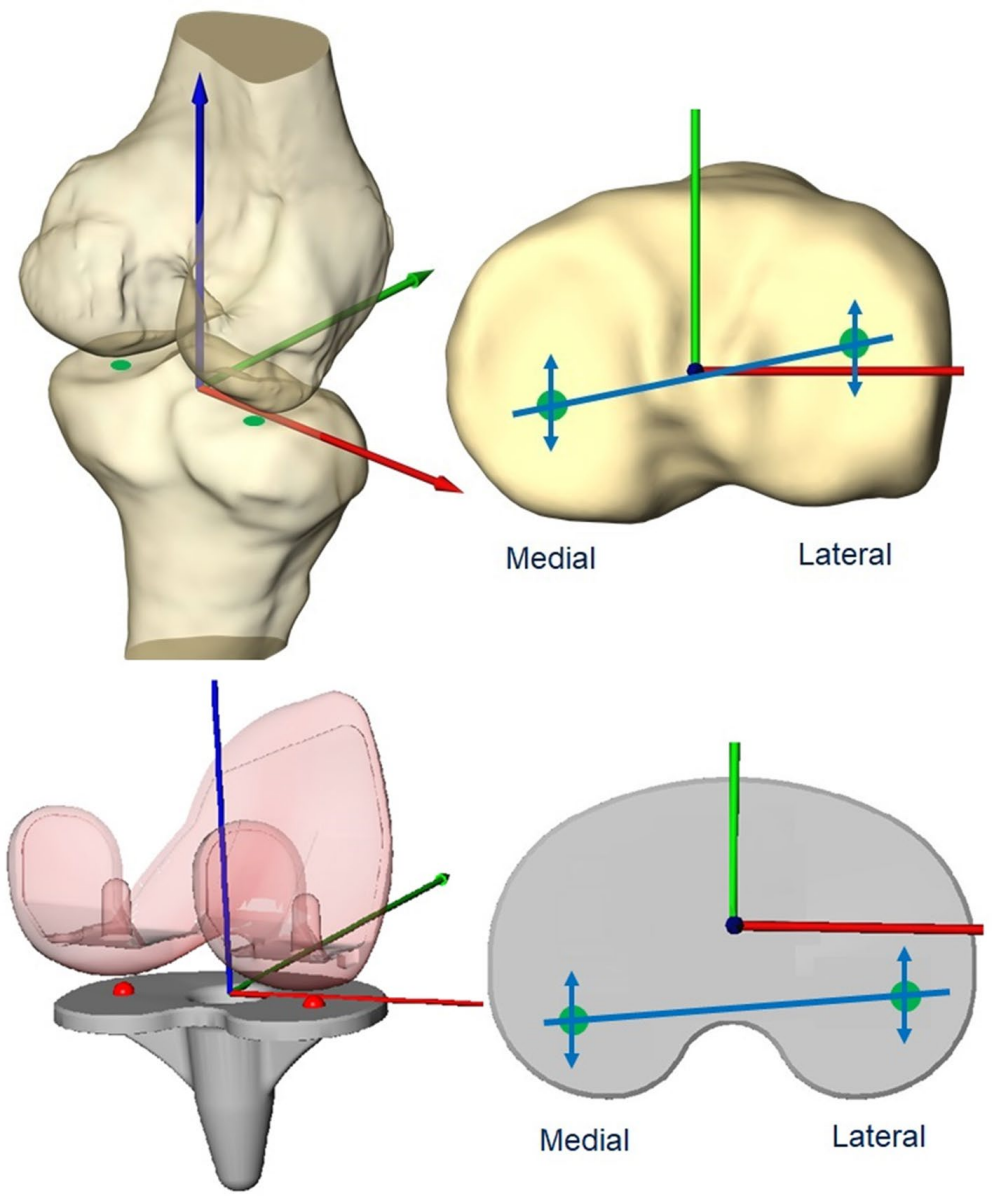
Determination of the anterior–posterior translation with the medial/lateral distal points. Top: example of episealer and healthy knees. Bottom: example of G- and J-Curve TKA components

Following the registration procedure, the positions and orientations of the femur and tibia were used to determine the most distal points of the lateral and medial femoral condyles. The most distal points were then projected onto the tibial plateau to generate a line (distal line) (Fig. 3) [19, 22].

The anterior–posterior (AP) translation, which is the main parameter of the present analysis, was expressed as the individual absolute position values of the medial and lateral distal points relative to the origin of the tibial coordinate system. Axial rotation was defined as the angle between the distal line and the medio-lateral tibial axis [19, 22]. To compare the outcome values between different trials at the same knee flexion angle, data were resampled using linear interpolation at the same 1° flexion increments.

### Institutional review board approval

This study was approved by the local ethics committee (Landesärztekammer-Brandenburg, Germany, approval number: S10(a)/2018) and registered at the German Clinical Trials Register (DRKS00020586). All subjects provided written informed consent.

### Statistical analysis

The data are presented as means and standard deviations. Normal distribution was tested using the Kolmogorov–Smirnov test. Mann–Whitney *U* tests were applied to the fluoroscopic data. Results were considered significant at an error probability of *p* < 0.05 using SPSS software (Version 22, IBM, Armonk, USA) [25].

A post hoc power analysis was performed to determine the sample sizes required to achieve statistical power of 1 − *β* = 0.80 and an alpha of 0.05. Using the magnitude of anterior–posterior translation as the main parameter based on previous investigations [18, 22], sample sizes of 10 × 10 for the comparison between episealer and healthy knees; 10 × 10 for the comparison between episealer and G-Curve TKA-treated knees; and 6 × 6 for the comparison between episealer and J-Curve TKA-treated knees were determined.

## Results

### Demographic data

Significant changes (*p* = 0.02) in passive flexion were observed between the pre- and post-operative state in the measured episealer subjects (Table 1). Compared to the healthy subjects, the episealer subjects were significantly older (*p* = 0.001), but were not different in terms of BMI (*p* = 0.14) and passive flexion (*p* = 0.06).

### Primary analysis (comparison episealer and healthy knees)

**Fig. 4:**
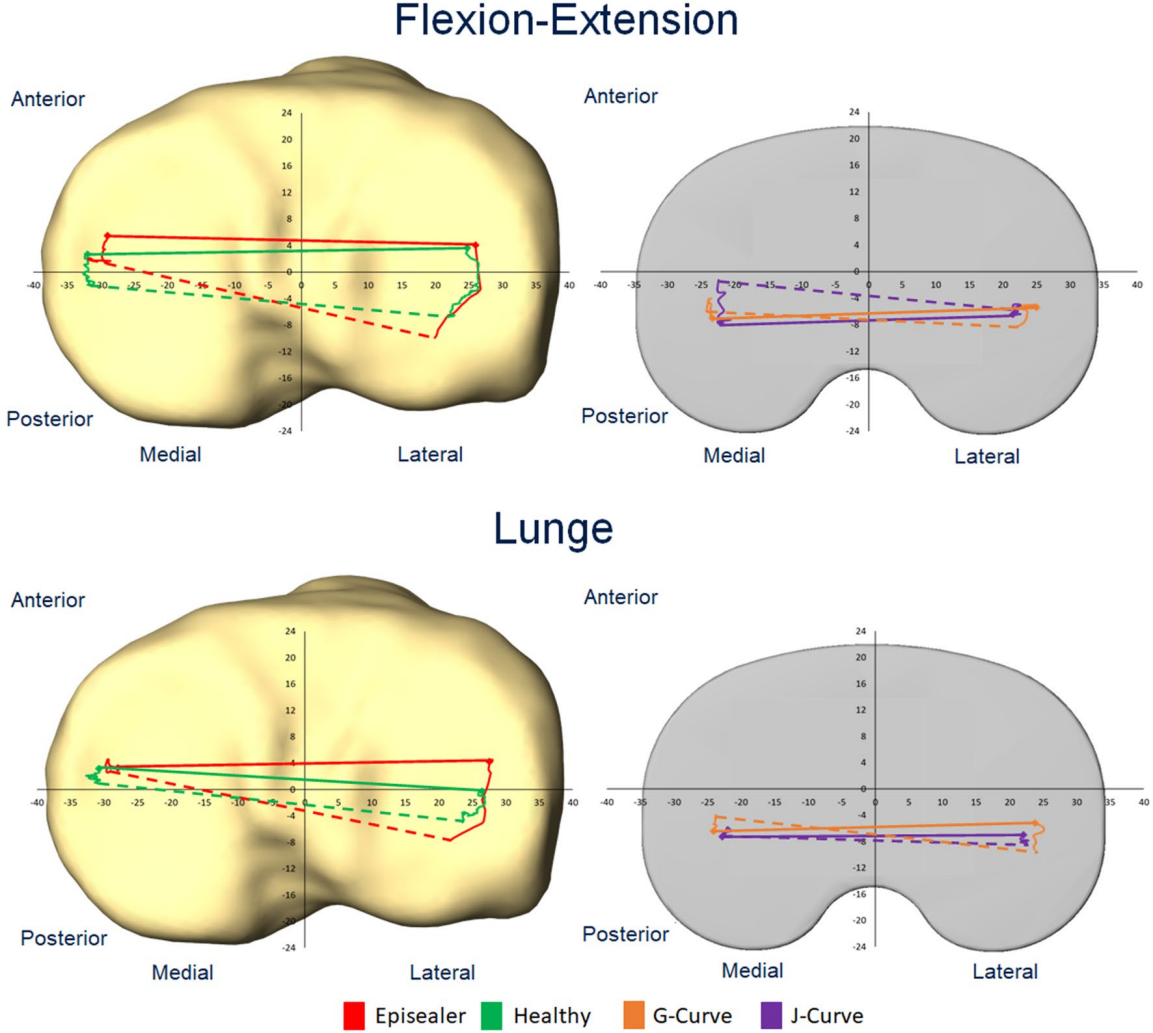
Left: absolute mean tibiofemoral kinematics during unloaded (flexion–extension) and loaded (lunge) activities during the main comparison between episealer and healthy knees. Right: absolute mean tibiofemoral kinematics during unloaded (flexion–extension) and loaded (lunge) activities during the secondary analysis of J-Curve and G-Curve TKA knees. Solid lines indicate the position of the distal points during knee extension. Dashed lines indicate the position of the distal points during knee flexion

During unloaded flexion–extension, the medial condyle in both the episealer knees and the heathy knees remained relatively stationary. The position of the medial distal points at maximal flexion was 1.6 ± 3.6 mm and − 0.8 ± 3.7 mm (*p* = 0.15) in the episealer and healthy knees, respectively. In the lateral compartment, a clear posterior position of the lateral condyle was observed in both the episealer (− 9.7 ± 3.5 mm) and healthy knees (− 6.1 ± 1.7 mm); however, it was significantly posterior (*p* = 0.02) in the episealer knees (Fig. 4). The relative values of this analysis, representing the magnitude of the movement, are presented in Table 2.

**Table 2:**
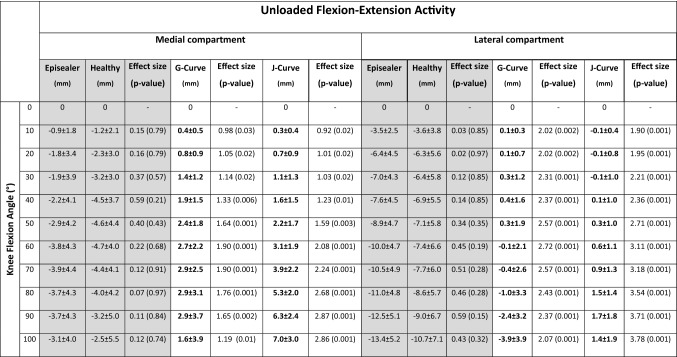
Means and standard deviations of the relative antero-posterior kinematics of the distal points during flexion–extension for the episealer and healthy knees (main comparison, gray background) and the TKA knees

Bold values indicate significant differences between episealer and healthy knees and between episealer and TKA knees. Effect size and *p* values for the respective comparison are also presented

This movement pattern resulted in progressive axial, external rotation of the femur relative to the tibia during the complete flexion cycle. This parameter was significantly higher (*p* = 0.001) in the episealer knees (13.6 ± 4.3°) compared to the healthy knees (7.0 ± 3.5°).

In the loaded activity, the medial condyle of the episealer and healthy knees remained consistently stationary with absolute positions at maximal flexion of 3.0 ± 3.0 mm and 2.0 ± 3.6 mm (*p* = 0.51), respectively. Similar to the unloaded activity, the lateral condyle translated posteriorly in both episealer and healthy knees (Table 3); however, it was in a significantly (*p* = 0.01) increased posterior position (− 9.9 ± 4.1 mm) in the episealer knees (Fig. 4).

**Table 3:**
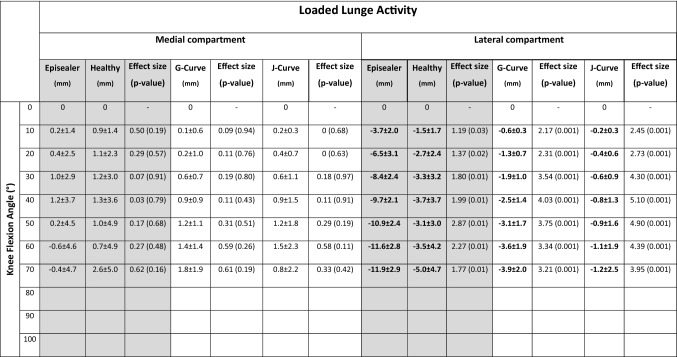
Means and standard deviations of the relative antero-posterior kinematics of the distal points during lunge for the episealer and healthy knees (main comparison, gray background) and the TKA knees

Bold values indicate significant differences between episealer and healthy knees and between episealer and TKA knees. Effect size and *p* values for the respective comparison are also presented

Absolute values for the external rotation were 11.7 ± 5.6° and 7.8 ± 5.3° (*p* = 0.06) in the episealer and healthy knees, respectively.

### Clinical data

At 12 months, pre-operative KOOS knee function scores of 44.1 ± 23.3 improved significantly (*p* = 0.001) to post-operative values of 85.9 ± 12.7. Also, significant improvement (*p* = 0.001) was observed for the VAS knee function assessment with pre-operative values of 6.1 ± 2.7 to post-operative values of 1.2 ± 1.2. EQ Health showed a significant improvement (*p* = 0.004) from pre-operative (65.0 ± 20.9) to post-operative (83.6 ± 13.2) values. The individual values can be found in Table 4.

**Table 4:** Values for the EQ 5D, VAS, and KOOS questionnaires

	EQ5D1		EQ5D2		EQ5D3		EQ5D4		EQ5D5		EQ health		KOOS		KOOSsym		KOOSpain		KOOSadl		KOOSsport		KOOSqol		VAS	
	Pre	Post	Pre	Post	Pre	Post	Pre	Post	Pre	Post	Pre	Post	Pre	Post	Pre	Post	Pre	Post	Pre	Post	Pre	Post	Pre	Post	Pre	Post
Episealer 01	2	1	1	1	2	1	2	1	1	1	75	80	26.8	83.3	42.8	82.1	25	83.3	32.3	94.1	0	55	12.5	75	7	1.3
Episealer 02/03	2	2	3	1	3	1	3	2	3	1	40	60	33.9	80.4	25	82.1	36.1	77.7	44.1	89.7	15	75	25	50	8.3	3.3
Episealer 04	2	1	2	1	3	1	3	1	2	1	90	90	2.4	99.4	14.2	96.4	0	100	0	100	0	100	0	100	9	1.3
Episealer 05	2	1	2	1	2	2	2	2	2	1	50	80	34.5	65.5	42.8	67.8	38.8	77.7	39.7	75	5	25	25	43.7	7	3
Episealer 06	1	1	1	1	1	1	1	1	1	1	90	95	80.4	95.2	92.8	96.4	86.1	100	89.7	98.5	40	70	56.2	100	0	0
Episealer 07	2	1	1	1	2	1	2	1	2	1	70	97	45.2	92.9	64.2	96.4	47.2	94.4	52.9	98.5	15	80	12.5	75	6	0.8
Episealer 08	2	1	1	1	1	1	2	1	1	1	70	100	50	99.4	60.7	100	47.2	97.2	58.8	100	20	100	37.5	100	6	0
Episealer 10	2	1	1	1	2	1	3	1	1	1	70	80	53.9	89.3	67.8	96.4	58.3	100	75	98.5	50	30	18.7	87.5	7.6	0
Episealer 11	2	2	1	1	3	2	2	2	1	1	30	70	69.6	67.9	71.4	78.5	83.3	69.4	88.2	73.5	30	50	6.25	43.7	4	1.1
Mean ± SD	1.9 ± 0.3	1.2 ± 0.4	1.4 ± 0.7	1 ± 0.0	2.1 ± 0.8	1.2 ± 0.4	2.2 ± 0.7	1.3 ± 0.5	1.6 ± 0.7	1 ± 0.0	65 ± 20.9	83.6 ± 13.2	44.1 ± 23.3	85.9 ± 12.7	53.5 ± 24.5	88.5 ± 11.1	46.8 ± 27.0	88.9 ± 11.9	53.4 ± 28.7	92 ± 10.6	19.4 ± 17.6	65 ± 27.3	21.5 ± 17.3	75 ± 24.0	6.1 ± 2.7	1.2 ± 1.2
*p* value (pre/post)	0.003		0.1		0.01		0.01		0.05		0.004		0.001		0.002		0.003		0.007		0.005		0.001		0.001	

Despite the small patient cohort, general significant improvement between the pre- and post-operative stages was noted

### Secondary analysis (comparison episealer and TKA knees)

During the unloaded flexion–extension activity, the absolute position at maximal flexion was − 0.6 ± 2.5 mm (*p* = 0.2) and − 5.2 ± 2.9 mm (*p* = 0.001) in the medial compartment and − 4.9 ± 2.7 mm (*p* = 0.01) and − 8.6 ± 3.2 mm (*p* = 0.42) in the lateral compartment for the J- and G-Curve TKA knees, respectively (Fig. 4). The displacement was characterized by increased anterior displacement in the medial compartment and reduced (*p* = 0.001) lateral rollback (Table 2). Coupled axial external rotation was significantly reduced compared to the episealer knees in both J- (5.1 ± 2.6°, *p* = 0.001) and G-Curve (4.0 ± 4.7°, *p* = 0.001) TKA knees.

In the loaded lunge activity, the absolute position at maximal flexion was − 5.6 ± 2.8 mm (*p* = 0.001) and − 4.7 ± 2.1 mm (*p* = 0.001) in the medial compartment and − 7.7 ± 2.4 mm (p = 0.09) and − 8.8 ± 2.2 mm (*p* = 0.44) in the lateral compartment for the J- and G-Curve TKA knees, respectively (Fig. 4). The displacement was characterized by a reduction in anterior displacement in the medial compartment and also in lateral rollback (*p* = 0.001) (Table 3). Coupled axial external rotation was significantly reduced in both J- (2.4 ± 5.9°, *p* = 0.001) and G-Curve (4.9 ± 4.6°, *p* = 0.001) TKA knees.

## Discussion

The most important finding of the present study was the nearly physiological knee joint kinematics during flexion observed in vivo in the episealer knees. Due to the reduced invasiveness of a partial, focal reconstruction, this study hypothesized that patient-specific resurfacing implants would lead to knee kinematics close to healthy knees, resulting in medial pivot and a high degree of femoral rollback during flexion.

Near complete absence of anterior shift was observed in the medial compartment during unloaded flexion–extension (Table 2, Appendix Fig. 5). Likewise, both episealer and healthy knees showed a similar extent of femoral rollback (Table 2, Appendix Fig. 6). Considering the different loading scenario during the lunge activity, a reduction of the anterior–posterior translation was expected due to increased axial load from the patient´s weight and muscle contraction. However, although a clear medial pivot (Table 3, Appendix Fig. 7) and lateral rollback (Table 3, Appendix Fig. 8) were observed in both episealer and healthy knees during the loaded lunge, the reduced lateral rollback observed in the healthy knees was not evidenced in the episealer knees, which showed a femoral rollback comparable in magnitude to the rollback during the unloaded activity.

The significantly higher lateral rollback during the lunge activity observed in the episealer knees may not be directly related to the episealer implant, but to possible post-operative muscle strength deficit. This deficit could result in an increase of femoral rollback due to insufficient stabilization at high flexion. Since specific electromyography analysis would be needed to corroborate this, the post-operative muscular deficit in episealer patients remain so far, an open question. However, post-operative muscle weakness has been reported previously in TKA patients [27].

Considering the patient-specific strategy in focal reconstruction of articular surfaces by the episealer system, it can be summarized that the minimal changes in the surrounding structures and ligament tensioning resulted in knee kinematics similar to those observed in a native knee joint. This was evidenced not only by the similar magnitude of movement at each compartment but also by the similar absolute condyle positions (Fig. 4) at extension and maximal flexion. Nevertheless, a certain degree of alteration was observed, which resulted in increased femoral lateral rollback that would be considered a moderate instability during the loaded lunge.

Coupled axial external rotation of the femur relative to the tibia in the episealer knees was present in both activities due to the effective and physiological mechanism of medial pivoting and lateral rollback. However, the magnitude was higher (significant during unloaded flexion–extension) than the one observed in the healthy knees. Considering the consistent medial pivoting observed, the increment in rotation can only be related to the higher lateral rollback and could be a consequence of the possible muscle weakness mentioned above.

Similar to previous analyses with resurfacing implants [10], and more recently with episealer knees [6], a significant increase in the VAS, KOOS, and almost all domains of EQ5D questionnaires was observed after 12 months, indicating clinical improvement. However, these results need to be interpreted carefully due to the limited number of knees analyzed.

Although not the main aim of the study, the secondary analysis of patients with TKA implants showed an expected contrast in knee joint kinematics. An anterior shift was noted in the J-Curve TKA group and to a certain extend in the G-Curve group (Table 2, Appendix Fig. 9). However, limited femoral rollback was observed in the G-Curve TKA cohort, probably due to the effect of the gradually changing sagittal femoral radius geometry (Table 2, Appendix Fig. 10). The effect of loading toward a stabilization of the anterior shift during loaded lunges was observed in the medial compartment, leading to similar stationary positions comparable to the episealer knees (Appendix Fig. 11).

Considering the large variability in surgical approaches in the implantation of traditional TKA designs as well as geometrical design constraints, this kinematic behavior was expected. The different kinematics should not be interpreted in detriment of established TKA procedures but more in terms of the achievement of understanding specific outcomes (Appendix Fig. 12).

This study is not without limitations. Although all episealer patients were recruited from a single center, operated by a single surgeon and treated under standardized protocols to guarantee homogeneity, the results must be interpreted cautiously due to the small number of knees measured. As specified in the “Methods” section, the recruitment process was affected by additional factors such as patient availability during the time of the study, concerns regarding X-ray assessment or lack of interest, resulting in only 9 of the initial 34 patients who completed the 12 month post-surgery measurements. Furthermore, pre-operative kinematic data were not available, which precludes a direct comparison of individual changes between the pre- and post-operative states.

The current results may help facilitate the decision-making process regarding the discrepancy around the treatment of patients in the GAP-age as well as possible prediction of kinematic outcomes. Despite positive results, careful pre-operative patient selection and clinical follow-up of treated patients are recommended for the long-term OA progression, particularly in the medial compartment of the proximal tibia. Moreover, further investigations are required not only in larger patient groups but also prospectively to assess pre- to post-operative kinematic changes. Analysis of additional activities such as walking and running would also offer valuable information regarding stability and changes in axial pivot. Such comparison could offer valuable knowledge on how reconstructive knee surgery could facilitate physiological knee kinematics and to what extent patients could benefit from such a resurfacing strategy compared to a partial or total reconstructive approach.

## Conclusion

At 12 month follow-up, a clear physiological knee kinematics pattern of medial pivot, lateral femoral rollback and coupled axial, external femoral rotation during flexion was observed in patients treated with an episealer resurfacing procedure. However, higher femoral rollback and axial external rotation in comparison to healthy knees were observed, suggesting possible post-operative muscle weakness and consequent insufficient stabilization at high flexion.

## Figures and Tables

**Fig. 5:**
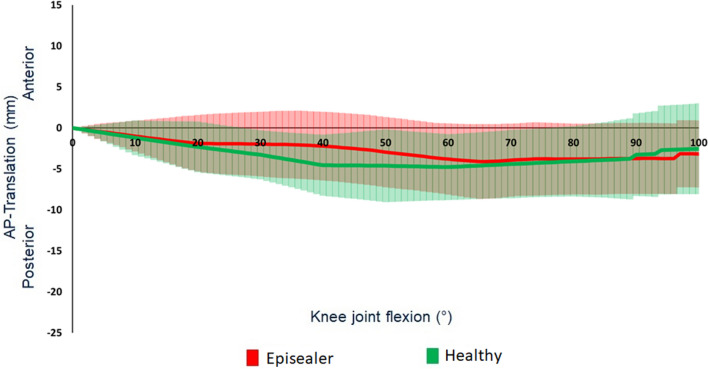
Relative kinematics during unloaded flexion–extension at the medial condyle. Comparison between episealer and healthy knees

**Fig. 6:**
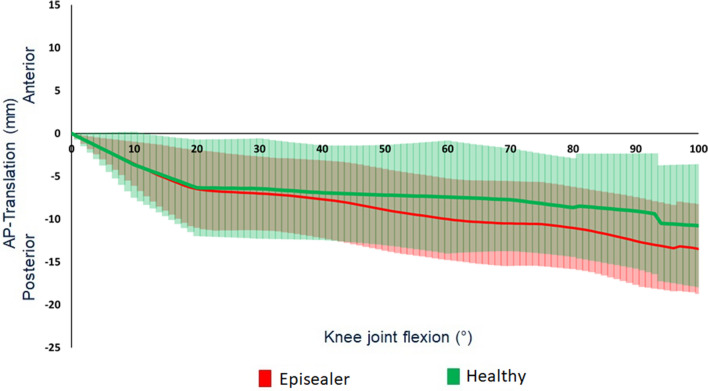
Relative kinematics during unloaded flexion–extension at the lateral condyle. Comparison between episealer and healthy knees

**Fig. 7:**
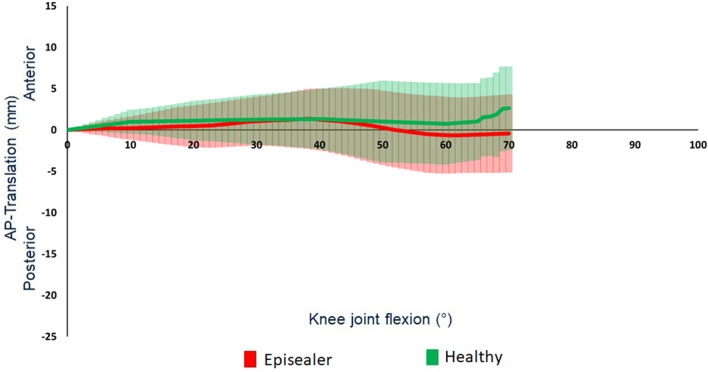
Relative kinematics during loaded lunge at the medial condyle. Comparison between episealer and healthy knees

**Fig. 8:**
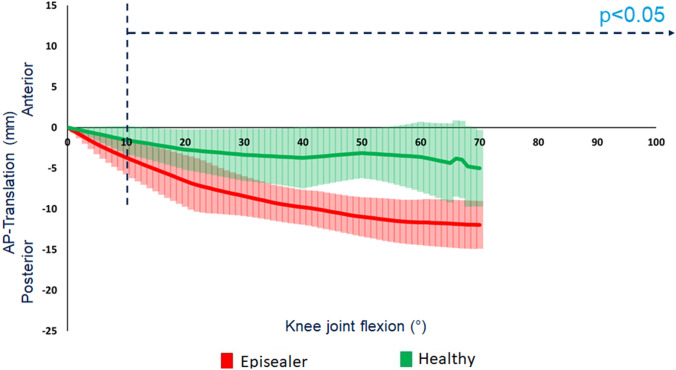
Relative kinematics during loaded lunge at the lateral condyle. Comparison between episealer and healthy knees

**Fig. 9:**
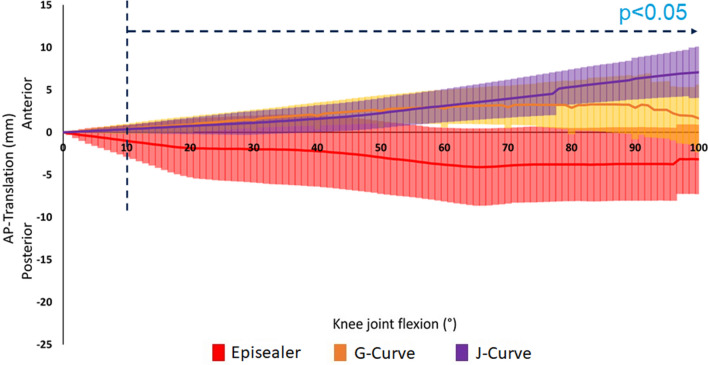
Relative kinematics during unloaded flexion–extension at the medial condyle. Comparison between episealer and TKA knees

**Fig. 10:**
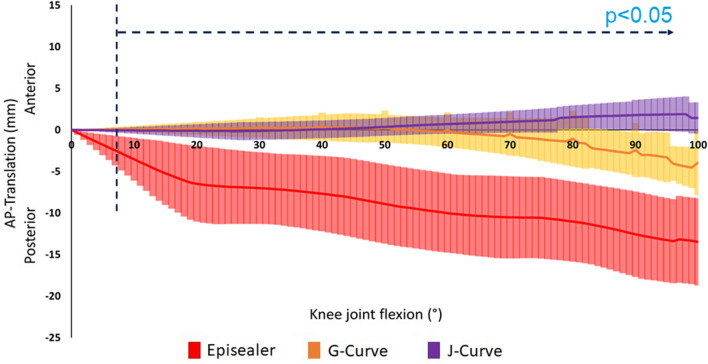
Relative kinematics during unloaded flexion–extension at the lateral condyle. Comparison between episealer and TKA knees

**Fig. 11:**
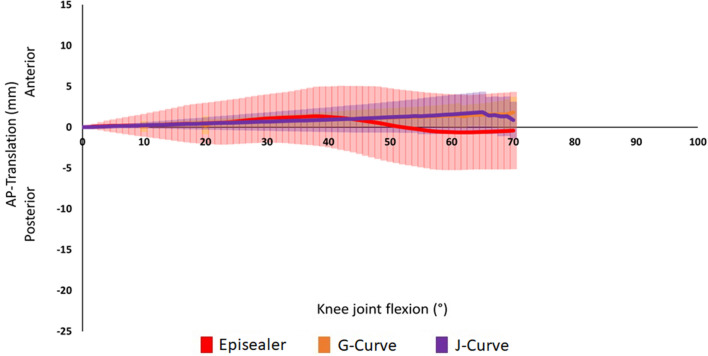
Relative kinematics during loaded lunge at the medial condyle. Comparison between episealer and TKA knees

**Fig. 12:**
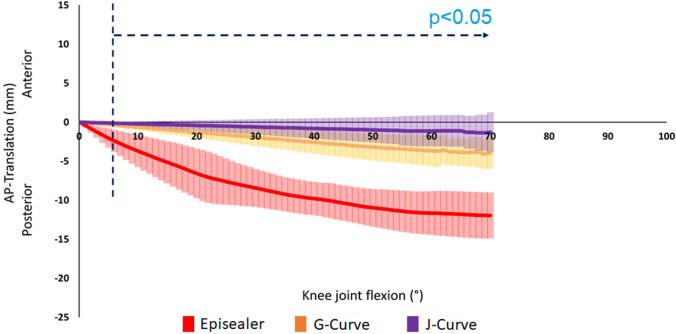
Relative kinematics during loaded lunge at the lateral condyle. Comparison between episealer and TKA knees

## Data Availability

The authors will make the data available upon reasonable request.

## Appendix

Figures 5, 6,7, 8, 9, 10, 11, 12 and Tables 5, 6.

**Table 5:** Comparison of the demographic data between the selected/analyzed episealer subjects and the non-selected episealer subjects

Selected	Sex	Age (years)	BMI	OD	Arth (cm)	P.M	Extension (pre) (°)	Extension (post) (°)	Passive flexion (pre) (°)	Passive flexion (post) (°)	Non-selected	Sex	Age (years)	BMI	OD	Arth (cm)	P.M	Extension (pre) (°)	Extension (post) (°)	Passive flexion (pre) (°)	Passive flexion (post) (°)
Episealer 01	m	52	30.6	4	9	No	0	0	120	130	Episealer 09	m	55	24.6	4	7	No	0	0	130	145
Episealer 02/03	f	48	21.9	4	7	No	0	0	130	130	Episealer 12	f	49	23.4	4	7	No	0	0	130	130
Episealer 04	f	46	22.6	4	7	No	0	0	100	120	Episealer 13	f	67	26.7	4	8	No	0	0	120	110
Episealer 05	f	57	25.9	4	7	No	0	0	130	130	Episealer 14	f	52	27.3	4	8	No	0	0	130	130
Episealer 06	f	73	26.0	4	8	No	0	0	110	130	Episealer 15	m	58	41.2	4	11	No	0	0	130	135
Episealer 07	m	54	36.1	4	10	No	0	0	130	130	Episealer 16	f	54	21.0	4	7	No	0	0	135	135
Episealer 08	m	59	32.3	4	11	No	0	0	130	130	Episealer 17	m	56	26.6	3	8	No	0	0	135	140
Episealer 10	m	56	38.9	4	10	No	0	0	120	130	Episealer 18	m	55	24.6	4	7	No	0	0	130	145
Episealer 11	m	61	29.3	3	9	No	0	0	130	140	Episealer 19	f	59	29.1	4	9	No	0	0	130	120
Mean ± SD		56.2 ± 7.9	29.3 ± 5.8						122.2 ± 10.9	130.0 ± 5.0	Episealer 20	m	51	24.7	3	7	No	0	0	130	130
*p* value (selected/non-selected)		0.55	0.53						0.40	0.56	Episealer 21	m	62	35.6	4	10	No	0	0	130	130
											Episealer 22	m	56	30.1	3	9	No	0	0	130	130
											Episealer 23	m	50	28.4	4	9	No	0	0	110	130
											Episealer 24	f	48	25.3	4	8	No	0	0	130	130
											Episealer 25	m	54	25.2	4	8	No	0	0	140	140
											Episealer 26	m	45	29.4	4	9	No	0	0	130	140
											Episealer 27	m	67	33.2	4	10	No	0	0	130	140
											Episealer 28	m	52	40.1	4	11	No	0	0	140	145
											Episealer 29	m	61	23.5	4	7	No	0	0	100	120
											Episealer 30	m	54	33.9	4	10	No	0	0	120	130
											Episealer 31	m	43	27.2	4	8	No	0	0	130	130
											Episealer 32	f	57	25.0	3	8	No	0	0	100	120
											Episealer 33	m	52	26.9	4	8	No	0	0	130	130
											Episealer 34	f	55	22.3	4	7	No	0	0	100	130
											Episealer 35	f	56	24.0	4	7	No	0	0	130	130
											Mean ± SD		54.7 ± 5.8	28.0 ± 5.2						126.0 ± 11.5	131.8 ± 8.5

Despite the factors described in the patient selection section that led to the non-recruitment of the subjects, non-significant differences were observed for age, BMI, and pre- and post-operative passive flexion

*OD* grade of osteochondral defects, *Arth* arthroscopy, *P.M.* patella maltracking

**Table 6:** Demographic data of the J-Curve and G-Curve TKA subjects

	Sex	Age (years)	BMI	OA grade	P.M	Extension (pre) (°)	Extension (post) (°)	Passive flexion (pre) (°)	Passive flexion (post) (°)		Sex	Age (years)	BMI	OA grade	P.M	Extension (pre) (°)	Extension (post) (°)	Passive flexion (pre) (°)	Passive flexion (post) (°)
J-Curve 01	f	69	37.8	4	No	0	0	95	130	G-Curve 01	m	67	28.1	4	No	0	0	90	115
J-Curve 02	m	73	24.9	4	No	0	0	100	130	G-Curve 02	m	70	33.3	4	No	0	0	95	130
J-Curve 03	m	65	32.2	4	No	0	0	100	140	G-Curve 03	f	73	27.3	4	No	5	0	110	130
J-Curve 04	f	74	29.3	4	No	5	0	90	125	G-Curve 04	f	75	35.0	4	No	0	0	100	140
J-Curve 05	f	67	36.1	4	No	0	0	110	125	G-Curve 05	f	55	29.7	4	No	0	0	95	130
J-Curve 06	f	78	23.9	4	No	5	0	110	130	G-Curve 06	f	69	33.8	4	No	0	0	110	125
J-Curve 07	f	67	31.2	4	No	0	0	100	125	G-Curve 07	f	59	44.3	4	No	5	0	95	105
J-Curve 08	f	68	28.9	4	No	0	0	90	120	G-Curve 08	f	56	23.8	4	No	5	0	100	125
J-Curve 09	f	63	51.2	4	No	0	0	95	115	G-Curve 09	m	72	27.3	4	No	0	0	100	120
J-Curve 10	f	56	29.1	4	No	0	0	110	140	G-Curve 10	f	78	30.5	4	No	0	0	95	115
Mean ± SD		67.9 ± 6.1	31.9 ± 7.8					100.0 ± 7.8	128.0 ± 7.9	G-Curve 11	f	57	30.2	4	No	5	0	110	130
*p* value (episealer post /J-Curve)		0.002	0.33					0.0005	0.52	G-Curve 12	m	76	30.6	4	No	0	0	110	140
										G-Curve 13	m	83	24.8	4	No	0	0	100	110
										G-Curve 14	f	54	26.1	4	No	0	0	90	120
										G-Curve 15	f	74	26.4	4	No	0	0	105	135
										G-Curve 16	m	70	40.2	4	No	5	0	110	130
										G-Curve 17	m	86	28.0	4	No	0	0	90	125
										G-Curve 18	f	57	42.5	4	No	0	0	90	125
										G-Curve 19	f	72	40.4	4	No	0	0	85	100
										G-Curve 20	f	78	30.2	4	No	5	0	100	130
										Mean ± SD		72.2 ± 9.6	32.1 ± 6.0					99.0 ± 8.0	124.0 ± 10.7
										*p* value (episealer post/G-Curve)		0.002	0.34					0.0006	0.12

In general, the TKA subjects were significantly older and had significantly reduced passive flexion pre-operatively but not postoperatively

## Abbreviations

OA: Osteoarthritis
UKA: Unicompartmental knee arthroplasty
TKA: Total knee arthroplasty
G-Curve: Gradually changing femoral radius
J-Curve: Conventional femoral multi-radius
VAS: Visual analog scale
KOOS: Knee Injury and Osteoarthritis Outcome Score
MRI: Magnetic resonance imaging
CR: Cruciate retaining
2D: Two-dimensional
3D: Three-dimensional
AP: Anterior–posterior

## Code availability

A custom code was used.
